# Beyond Difference Scores: Unlocking Insights with Polynomial Regression in Studies on the Effects of Implicit-Explicit Congruency

**DOI:** 10.5334/pb.1246

**Published:** 2024-04-05

**Authors:** Liselotte Visser, Ron Pat-el, Johan Lataster, Jacques Van Lankveld, Nele Jacobs

**Affiliations:** 1 Open University Heerlen, The Netherlands

**Keywords:** polynomial regression, response surface methodology, self-esteem, discrepancy scores, psychopathology

## Abstract

The aim of our study was to investigate whether theories of congruence are better tested using polynomial regression analysis, rather than expressing discrepancy between implicit and explicit measures as continuous or categorical difference scores. This paper also aims to make knowledge more accessible by providing a step-by-step explanation of both methods, illustrating differences between them, and making materials openly available for other researchers. In this paper, implicit and explicit measures of self-esteem are used as predictors for depressive symptoms, anxiety, and aggression in a general population sample (*N* = 135). Explicit self-esteem was measured using the Rosenberg Self-Esteem Scale, implicit self-esteem was measured using the Implicit Association Test, and the Symptom Questionnaire was used to measure depressive symptoms, anxiety, and aggression. The results show those difference score models all imply that the discrepancy between implicit and explicit self-esteem explains depression and anxiety, but not aggression. However, polynomial regression analysis shows that depression and anxiety are not accounted for by the explicit-implicit discrepancy as such, but are foremost explained by explicit self-esteem. Polynomial regression has the potential to evaluate more complex and more detailed hypotheses than what would be possible using statistical approaches based on discrepancy scores. It is therefore recommended for future research aimed at disentangling the roles of explicit and implicit self-esteem in psychological outcomes.

## 1 Introduction

Individuals with low self-esteem think and behave in ways that diminish their quality of life (Swann Jr et al., 2007), whereas high self-esteem predicts success and well-being in life domains of relationships, work, and health (Orth & Robins, 2014). Positive psychology, building on Maslov’s conception of self-actualization has long posited that the self-valuing process is dependent on the quality of the self-concept (Ryan & Deci, 2000). Central to positive psychology’s paradigm is the position of Rogers who believed that for a person to achieve self-actualization they must be in a state of congruence (Rogers, 1957). The idea is that self-actualization occurs when a person’s “ideal self” (i.e., whom they would like to be) is congruent with their actual behavior (self-image). The closer self-image and ideal self are to each other, the more consistent or congruent the person is which increases self-esteem.

The interplay between implicit and explicit manifestations of one’s own self-esteem is often explained from the perspective of dual-process theory. Dual-process theory (Evans, 2008; Kahneman & Frederick, 2005) states that information is processed along two distinct pathways, a deliberate (explicit) and an automatic (implicit) pathway. The implication of this theory in the context of self-esteem would be the existence of two different self-esteem dimensions that play distinct roles in controlled and automatic behaviors: explicit self-esteem and implicit self-esteem (Buhrmester et al., 2011; Gawronski & Bodenhausen, 2006; Spalding & Hardin, 1999; Strack & Deutsch, 2004). Explicit self-esteem is thought to represent a conscious, cognitive and controllable reflection on one’s own self-worth (Schröder-Abé et al., 2007). Implicit self-esteem, on the other hand, prevails in situations in which the individual does not have particular goals, has little time to think, and/or is not aware of the outcome of the cognitive process (De Houwer et al., 2009). Implicit does not necessarily mean unconscious: participants may be aware of the attribute being assessed, but they have minimal control over the measurement outcome (Steffens, 2004). Kernis (2003) hypothesized that when implicit and explicit self-esteem differ, the direction of the discrepancy changes its meaning. When low implicit self-esteem concurs with high explicit self-esteem it is referred to as fragile self-esteem, whereas the combination of high implicit self-esteem with low explicit self-esteem is referred to as damaged self-esteem. Several studies that explored the effects of the discrepancy between explicit and implicit self-esteem have shown associations of discrepancy-size with depression (Creemers et al., 2013; Creemers et al., 2012; Franck et al., 2007; Leeuwis et al., 2014; Valiente et al., 2011), anxiety (Rudman & Ashmore, 2007; Schreiber et al., 2012; van Tuijl et al., 2014) and aggression (Jordan et al., 2005; Sandstrom & Jordan, 2008; Schröder-Abé et al., 2007).

Dual-process theory has met with well-reasoned critique in the past decade, mainly because of concerns with the idea that the two systems are sufficiently independent. Keren & Schul (2009) provide a thorough summary of key critical points, and assert that testing the theory of dual-process requires a re-evaluation of research methodologies used in studies that assume dual-processes take place. These critical points are sufficiently meaningful to warrant our attention. For example, in the above-mentioned studies, implicit and explicit pathways were operationalized as a discrepancy score. The discrepancy score was operationalized as a difference score between explicit and implicit self-esteem, representing the degree and direction of congruence between explicit and implicit self-esteem. However, difference scores are rife with methodological problems, often resulting in misleading conclusions. Edwards (2001) outlined many of the problems with difference-scores, like reliability issues, reduced effect sizes and an increased risk of errors. One such problem is the attenuation of error in difference scores; the reliability of each component part is attenuated by the correlation between them (Cohen & Cohen 1983), which means that the reliability of a difference score measure is usually less than the reliability of each component.

One primary concern associated with difference scores is their tendency to reduce true score variance (May and Hittner, 2003). True score variance reflects the variability in scores attributable to actual differences in ability or attitudes, while error variance encompasses variability introduced by random factors, such as measurement error. When calculating a difference score like d600, the true score variance is diminished by an amount corresponding to the square of the correlation between the pretest and posttest scores. This reduction occurs because the difference score is sensitive only to changes in ability or attitudes that are shared between the pretest and posttest measurements. Consequently, the reliance on the common variance may compromise the reliability of difference scores compared to the individual reliability of pretest and posttest scores. In addition, as in the above mentioned studies, implicit and explicit self-esteem are fundamentally differenced constructs and the method of their measurement necessarily imply that they are conceptualized and operationalized in different ways, which affects reliability and content validity, especially when these two constructs are directly compared to each other. Self-report scales of Self-Esteem often show a higher test-retest correlation and a lower situational variability compared to implicit measures, which means that explicit measures tend to be more stable over time compared to implicit measures. (Dentale et al., 2019). The IAT variable is susceptible to errors due to its reliance on reaction times, which can be affected by various factors like fatigue, concentration, and motivation. In contrast, the Rosenberg Self-esteem is a questionnaire specifically crafted to yield a dependable and valid assessment of explicit self-esteem. Dentale et al.’s (2019) findings imply the presence of residual noise in the discrepancy score between explicit and implicit self-esteem. This is attributed to the inherent error-proneness of the IAT variable, whereas the Rosenberg Self-esteem, being an explicit measurement, is not prone to such errors.

Perhaps most importantly, the use of difference scores implies an intuitive, but specific, and restrictive hypothesis, namely that the components of the difference have equal but opposite effects (Edwards, 1994). This model might be a poor reflection of the underlying reality, and several alternative models for difference scores that are often better able to describe the relationship between components have been proposed (Humberg et al., 2018; Mota et al., 2020). One important problem with the model underlying the classic use of difference scores is that it obfuscates the relative weight of each component in relation to a criterion variable, i.e. it hides whether both factors pull equal weight, or if one variable is doing most of the legwork. For example, a significant association between a difference score (such as the difference between explicit and implicit self-esteem) and an outcome measure (such as depression) may reflect only a main effect of one of the core variables (explicit self-esteem or implicit self-esteem) and not per se the effect of the degree of discrepancy. In the extreme case that one of these variables has zero effect on the criterion, the use of a difference score amounts to little more than centering the main effect to zero with a random residual deviation, turning the difference score into a worse predictor than the main effect would have been in itself.

To address methodological challenges, alternative approaches have been proposed in the literature. Edwards (1993) drew inspiration from surface response methodology and advocated for the utilization of polynomial regression equations to explore the intricate interplay between component parts in a difference score concerning a criterion variable. Edwards’s (1993) method involves eschewing the use of difference scores altogether and constructing polynomial regression models with each component as a separate predictor. This enables the assessment of the relative importance of each component. The incorporation of polynomials facilitates the depiction of a curved surface, describing the relationship between components and the criterion. This approach allows for the examination of more complex relationships that are beyond the scope of traditional difference scores.

In a subsequent work, Edwards (1994) delves into two specific congruency hypotheses within the realm of RSA, articulated as the X = Y and X = –Y lines. The X = Y line epitomizes perfect congruence, signifying an ideal alignment between two constructs. Conversely, the X = –Y line represents the extreme of incongruence, where the two constructs are diametrically opposed, leading to maximal incongruence and potential negative outcomes such as job dissatisfaction, stress, and turnover. These extremes serve as crucial benchmarks for testing congruency-related hypotheses directly through RSA.

For instance, if the observed congruence score aligns closer to the X = Y line than the X = –Y line, it implies a positive relationship between congruence and the outcome. In this scenario, an increase in congruence corresponds to an increased likelihood of a positive outcome. Conversely, if the observed congruence score is closer to the X = –Y line than the X = Y line, it suggests a negative relationship between congruence and the outcome. Here, an increase in congruence is associated with a decrease in the expected outcome.

Rather than solely examining the effect of the difference on a criterion, the combination of polynomial regression with response surface methods enables the testing of more nuanced hypotheses. By analyzing the curvature along the X = Y and X = –Y lines, one can empirically test (instead of assuming) classic difference score hypotheses, such as whether (a) each component part carries equal weight, (b) the criterion is optimized at a constant value when the two components are equal, and (c) the criterion changes systematically when deviating from these congruency lines. Therefore, the primary advantages of polynomial regression lie in its ability to test a priori hypotheses derived from theory, its resistance to attenuated reliability, and its capacity to determine the contribution of each component part in predicting a criterion.

In short, these equations get around many problems that come along with the use of difference scores and at the same time allow to draw valid conclusions about congruence effects. Nevertheless, polynomial regression in lieu of difference scores is not common practice, probably because the results of such equations are not so easy to interpret. The polynomial models and their constraints can be quite arcane to anyone not already familiar with advanced calculus, and disentangling the unique and joint effects expressed by main effects, squared main effects and interaction terms is challenging. It is more practical to plot the polynomial model as a 3-D plot, a response surface, either accompanied by a contour plot or by using colours to express the height of the surface. In addition, polynomial regression is not without limitations. This approach is only useful when the differences between variables are the predictors, and a different approach is needed when discrepancy is the dependent variable (Edwards, 1995). Polynomial regression is susceptible to outliers given that it squares the main effects, which has an exponential effect on outliers, requiring rigorous data screening.

There are two research questions here that need to be addressed. The first question is: Should implicit and explicit self-esteem be merged in one variable (difference scores) or not? The second question is: Should the effect of self-esteem (either difference scores, either the two original variables) be modelled as polynomial? The main goal of this study is to demonstrate polynomial regression as an approach that allows to investigate the associations between explicit self-esteem, implicit self-esteem and respectively depression, anxiety and aggression through specific hypotheses derived from fit patterns. We will provide an empirical example to demonstrate the differences between the approaches, and how to interpret their results. In the first approach, numerical congruence as expressed in a difference score between explicit and implicit self-esteem will be used. In the second approach, polynomial regression equations will be applied in order to analyse fit patterns based on the relative importance of each predictor, but also the shape of the curved surface, and meaningful axes along these shapes. The overall aim of this paper is to illustrate the difference in results and conclusions, depending on the approach that is used, and why it is important to move away from numerical congruence (i.e. difference scores) as predictors, especially in research concerning the congruence between explicit and implicit constructs. This paper also aims to make knowledge more accessible by providing a step by step explanation of both methods, illustrating differences between them, and making materials openly available for other researchers.

In the method section the empirical example will first be introduced and explained. In the Analysis subsection a more detailed explanation will be given regarding Polynomial Regression, such as the specific hypotheses that can be tested, how to interpret the 3D representation of the model, the most important axes in the model and their interpretation. The results section will demonstrate the outcomes from the two approaches and their differences will be discussed in the discussion section. The purpose of this paper was to be easy to understand for researchers who have little background in advanced calculus or linear algebra. For this reason both the methods and the introduction include little to no equations and proofs, unless absolutely necessary. In the appendix we will offer the R-code that was used to conduct the polynomial regression analyses, as well as a mathematical explanation for those interested in it.

## 2 Disclosure

The study is not preregistered. This study involved an analysis of existing data rather than new data collection. All simulations and other analyses conducted are reported. Participation in the study was voluntary and participants gave digital informed consent after being fully informed about the study, and having had the opportunity to have any questions answered. The study was carried out in accordance with The Code of Ethics of the World Medical Association (Declaration of Helsinki) for medical research involving humans. Information regarding all procedures applied and all measures assessed in this study as well as the data and the data analysis scripts needed to reproduce the results are openly accessible: https://osf.io/q7c5f/.

## 3 Method

### 3.1 Participants

Convenience sampling was used to recruit participants within the social networks of psychology students of the Open University of the Netherlands. Participants were included if they were over 18 years of age and were fluent in the national language. The sample (N = 135) consisted of 60 male (44.4%) and 74 female participants (54.8%) with an age range of 19 to 77 years (M = 44.6; SD = 11.45). Of one participant no demographic data were available. Forty three participants (31.9%) had completed secondary education or lower, 44 (32.6%) had completed undergraduate education, and 47 (34.8%) had completed an academic degree or higher. Most participants (n = 111, 82.2%) were employed, and 111 participants (82.2%) were in a relationship. This sample meets the power requirement for second-order polynomial regressions with seven parameters (Khamis & Kepler, 2010).

### 3.2 Materials and Procedure

The constructs were measured in several phases. First participants were asked to complete online questionnaires in which the dependent variables and explicit self-esteem were measured. After completion participants completed the implicit self-esteem measurement which was repeated two weeks later.

#### 3.2.1 Depressive symptoms, anxiety and aggression

The Symptom Questionnaire (Carlier et al., 2012) is a validated screening tool for psychopathology. Four subscales cover different aspects of psychopathology: depression, anxiety, somatization and agoraphobia. In addition, five subscales assess specific aspects of problems in behaviour and/or functioning: aggression, cognitive problems, social phobia, work and vitality/optimism. Respondents were asked to indicate what answer best applied to them, taking the past week as the reference period. Each item was rated by the respondent on a 5-point Likert-scale from 0 (“never”) to 4 (“very often”). Scores were added to form a sum-score for each subscale. For the current study, the Depression subscale (MOOD), the Anxiety subscale (ANXI) and the Aggression subscale (AGGR) were selected. Depressive symptoms were measured using 6 items (e.g. “I struggled to get the day started”), anxiety was measured using 6 items (e.g. “I felt jittery and nervous”) and aggression was measured using 4 items (e.g. “I struggled to control my anger”). Internal consistencies of the items per subscales were high with Cronbach’s *α* = .87 (MOOD), and .89 (ANXI), to Cronbach’s *α* = .76 (AGGR), which were similar to the reliabilities reported by Carlier et al. (2012).

#### 3.2.2 Explicit self-esteem

The Rosenberg Self-Esteem Scale (Rosenberg, 2015) was used to measure explicit self-esteem operationalized as global self-esteem using 10 items. Responses were given on a 4-point scale ranging from 0 (“strongly disagree”) to 3 (”strongly agree”). The questionnaire contains five positively worded items (e.g. “On the whole, I’m satisfied with myself”) and five negatively worded items (e.g. “At times I think I am no good at all”). The scores on the latter items were recoded such that higher scores reflected higher self-esteem. The scores were summed to represent explicit self-esteem. The reliability of the RES was high with Cronbach’s *α* = .89.

#### 3.2.3 Implicit self-esteem

A Single-Target Implicit Association Test (IAT) was used to measure implicit-self-esteem. The purpose of an IAT is to indirectly measure a construct by using the relative strength of the association between two pairs of concepts related to the construct (Greenwald et al., 1998). Participants were asked to sort stimuli representing four concepts into two response categories (termed ‘target’ categories and ‘attribute’ categories), each of which included two of the four concepts. When two concepts, sharing the same response key, are strongly associated, the sorting task is assumed to be easier than when the two concepts are either weakly associated or bipolar-opposites (Greenwald et al., 2003), and consequently the pertinent reaction time is assumed to be lower. The difference in response time reveals the automatic reaction towards a target object, in this case the self. The Single-Target Implicit Association Test (ST-IAT) measures the evaluation of a target object without the need to simultaneously evaluate a counter-category as in the original Implicit Association Test (Bluemke & Friese, 2008).

Following Greenwald et al. (1998) the Self-Esteem IAT in the current study consisted of five blocks in which participants had to categorize words into different categories – of which the labels were continuously presented in the left and right upper corners of the screen – by pressing a left (z) or right (m) response button. To represent the “Myself” target, the Dutch words for “me,” “myself,” “I,” and “mine” were used. Stimulus words for low self-esteem were the Dutch equivalents of “bad,” “inferior,” “failed,” and “dissatisfied”. Stimulus words for high self-esteem were the Dutch equivalents of “good,” “successful,” “equal,” and “valuable”. Block 1 consisted of 20 trials using the left key for “low self-esteem” and the right key for “high self-esteem”. Block 2 consisted of 15 trials using the left key for “myself” and “low self-esteem” and the right key for “high self-esteem”. Block 3 consisted of 45 trials using the left key for “myself” and “low self-esteem” and the right key for “high self-esteem”. Block 4 consisted of 15 trials using the left key for “low self-esteem” and the right key for “myself” and “high self-esteem”. Block 5 consisted of 45 trials using the left key for “low self-esteem” and the right key for “myself” and “high self-esteem”. The response time for each trial was determined by measuring the elapsed time between stimulus onset and keypress.

Implicit self-esteem was operationalized through the IAT using the D600 scoring algorithm recommended by Greenwald et al. (2003). The experiment consisted five blocks, the first of which was a tutorial block, and block 2 and 4 were practice blocks. The data of block 3 and 5 (test trial blocks) were used in the analysis. As recommended by Greenwald et al. (2003), only reaction times in the range of 400–2500 ms were kept in the study. All reaction times (RT) in the range of 2500 – 10,000 ms were replaced with a RT of 2500 ms (to wit, in block two 46 of the 2025 values, in block three 46 of the 6075 values, in block four 36 of the 2025 values and in block five 34 of the 6075 values).

Latencies above 10,000 ms were excluded before any further computations (to wit, 1 of the 2025 values in block two, 5 of the 6075 values in block three, 1 of the 2025 values in block 4 and 0 of the 6075 values in block 5), following Greenwald et al. (2003). After this first phase of replacement and deletion the reaction times for error trials were replaced with the mean RT of the correct responses in the same block plus a 600 ms penalty, also following Greenwald et al. (2003).

For the implicit measurement of self-esteem, the D600 index score was calculated as the difference between the mean RTs to the “myself + low self-esteem” and the “myself + high self-esteem” combination blocks, divided by the standard deviation calculated across all blocks except the attribute practice block. Thus, positive IAT scores reflected high implicit self-esteem, whereas negative scores reflected low implicit self-esteem.

The internal consistency was calculated by measuring the split-half reliability by correlating IAT D600 scores of the even trials with the odd trials within each block. The test-retest reliability was established with the Pearson correlation between the baseline measurement and the second measurement two weeks later. The split-half reliabilities of the first measurement of block 1 to 5 of the IAT were respectively Rs = .94, Rs = .90, Rs = .94, Rs = .91, Rs = .94. The test-retest reliability coefficients of blocks 1 to 5 of the IAT for the first and second measurement were respectively *r* = .63, *r* = .67, *r* = .78, *r* = .55, and *r* = .77 (all *p*s < .01).

### 3.3 Data analyses

Four different approaches were used to demonstrate the accordance and discordance in the results and conclusions between the different approaches. Regardless of the approach, in order to examine the associations between implicit and explicit self-esteem on the one hand, and depressive symptoms, anxiety and aggression on the other, hierarchical multiple regression analyses were performed. Prior to analyses the implicit and explicit self-esteem scores were standardized to help their interpretation. The four analyses will be outlined in the next subsections.

#### Analysis 1: Continuous Difference Scores

The self-esteem discrepancy was first computed as the difference between the standardized scores of explicit and implicit self-esteem. The difference score was calculated by subtracting the implicit (IAT) score from the standardized explicit (Rosenberg SE) score, and entered into a linear regression model. The regression model can be expressed as:


1 \[
\hat Y = {\beta _0} + {\beta _1}X + \epsilon
\]

Here, *Y* ^ is the predicted dependent variable, β_0_ is the intercept, β_1_ is the coefficient for the self-esteem discrepancy, X, and ϵ represents the error term.

In a linear difference score model it is possible to add polynomials such as quadratic terms to model more complex patterns of difference scores. An example quadratic relationship can be expressed as:


2 \[
\hat Y = {\beta _0} + {\beta _1}X + {\beta _2}{X^2} + \epsilon
\]

Where *β*_2_ is the coefficient for the square of the difference score *X*^2^, which models the effect of the difference score on the dependent variable as a parabola.

In the continuous approach it is difficult to test specific hypotheses regarding the exact effect of the type of difference (negative or positive) on the dependent variable. Rather, the direction and strength of the association are used to ascertain whether a positive or negative difference score is positively related to the outcome variables and to what degree.

#### Analysis 2: Categorical Difference Scores Some

researchers believe that the problems with continuous difference scores can be solved by reducing the measurement-level of the difference score by creating subgroups based on the congruence between two component measures (e.g. Mersman & Donaldson, 2000). This belief is wrong (e.g., Edwards, 2001) and subgroup Polynomial regression’s foremost goal is to test hypotheses derived from congruenceing adds new problems to the difference score measure without alleviating the old ones, such as the attenuation of error in difference scores, the proneness of the implicit measurement to contextual factors, residual noise in the difference scores and the obfuscating of the relative weight of each component in relation to a criterion variable. The main problem with subgrouping is that it still remains a difference score, albeit one which now carries less information and reduced explained variance. To demonstrate the subgrouping approach the difference score between explicit and implicit self-esteem was split into three groups based on Kernis (2003) classification. Respondents with difference scores 0.5 SD below zero were classified as *fragile* (combination of low implicit self-esteem with high explicit self-esteem) and respondents with difference scores 0.5 SD above zero were classified as *damaged* (high implicit self-esteem combined with low explicit self-esteem). The remaining respondents were classified as *congruent* in explicit and implicit self-esteem. These three groups were subsequently split into two dummy variables representing fragile and damaged self-esteem, in order to compare their regression weights to the congruent group. These dummies were entered into a linear regression model. In the categorical approach it is slightly easier than in the continuous case to test specific hypotheses regarding the effect of the type of difference (negative or positive) on the dependent variable. The regression equation for this model is expressed as:


3 \[
\hat Y = {\beta _0} + {\beta _1}{X_{damaged}} + {\beta _2}{X_{fragile}} + \epsilon
\]

Here, *Y* ^ is the predicted dependent variable, *β*_0_ is the intercept, *β*_1_ and *β*_2_ are the coefficients for the dummy variables *X_damaged_* and *X_fragile_* respectively, and ϵ represents the error term. In this model the intercept takes on the value of the mean of the ‘congruent’ category, and the coefficient for the dummy variables denote the deviation from this mean.

One critique of the continuous approach can be that even though the explained variance is retained by using the high measurement-level, the interpretation of the regression weights amounts to a subgrouping approach without a congruent group, i.e. everyone is classified as incongruent in some way. In contrast, the categorical approach involves a user-defined range of difference scores that are deemed congruent scores, with tails in the distributions of the difference score serving as incongruent scores. Incongruency in the continuous and categorical approach in effect are modelled differently, as the categorical approach foregoes the less extreme deviances from perfect congruity. The categorical approach, by categorizing a range of scores as congruent and focusing on relatively extreme departures, introduces a trade-off between increased power for detecting extreme incongruencies and potential loss of sensitivity to smaller departures from congruence.

Just like in the continuous approach, the direction and strength of the association of the dummy variables are used to ascertain whether a positive or negative difference score is positively related to the outcome variables and to what degree. The regression weights are the mean differences of the subgroups relative to the intercept, which is the mean of the congruent group.

#### Analysis 3: Mixed Categorical and Continuous

In addition to the classical difference score approaches outlined above we will demonstrate the mixed approach followed by Creemers et al. (2012), who propose a method that splits a continuous difference score into two variables: (1) The absolute difference between the standardized score on implicit and explicit self-esteem, to indicated the size of discrepancy, i.e. a continuous difference score with only positive values; and (2) a dichotomous variable that indicates the direction of the discrepancy between implicit and explicit self-esteem (implicit < explicit or implicit > explicit). Their method makes no explicit allowance for respondents with congruent implicit and explicit scores. The size of the discrepancy (continuous) and the direction of the discrepancy (dichotomous) were entered in a hierarchical regression in step 1 and their interaction in step 2. The regression model is expressed as:


4 \[
\hat Y = {\beta _0} + {\beta _1}{X_{fragile}} + {\beta _2}{X_2} + {\beta _3}{X_{fragile}}{X_2} + \epsilon
\]

Here, *Y* ^ is the predicted dependent variable, *β*_0_ is the intercept, *β*_1_ is the coefficient for the dummy variable *X_fragile_*, which functions as a direction variable which is attained through dummy coding: a value of 0 for damaged self-esteem and 1 for fragile self-esteem. *β*_2_ is the coefficient for continuous dependent variable X_2_, which represents the size of the difference. *β*_3_*X_fragile_ X_2_* represents the interaction between the direction and size, and ϵ represents the error term. In this model the intercept takes on the value of the mean of the ‘damaged’ category, and the coefficient for the dummy variable denotes the deviation from this mean.

#### Analysis 4: Response Surface Analysis and Polynomial Regression

RSA (Response Surface Analysis) is a statistical technique used to study relationships between multiple independent variables and a response variable. RSA often involves modeling the response surface using mathematical models, and these models may include polynomial terms to capture non-linearities. Polynomial Regression is a specific type of regression analysis focused on modeling relationships using polynomial functions.

Difference score theories have some key similarities to this original goal of RSA. The theory behind difference scores is that congruent values on the variables (i.e., no difference between explicit and implicit) optimize a response. For example, one could hypothesize that individuals are most happy, or are least depressed when implicit and explicit self-esteem are perfectly congruent. The problem, which RSA solves, is that the theory underlying difference scores is severely restrictive. Most important among its many restrictions, is the assumption that perfect congruence optimizes the response. ‘Optimize’ here implies a global optimum (the best of all possible solutions, e.g., highest possible happiness, or lowest possible sadness) rather than a local optimum (the best in a particular neighbourhood of solutions). By visualizing the 3D response surface the researcher is able to visually determine the way in which the implicit and explicit measures affect the response variable, if, and if so where, it optimizes the response. To make these determinations it is important to be familiar with the following key terms in RSA.

Stationary Point. The global optimum, either the overall minimum or maximum, of the surface is called the stationary point. It is the point where the slope of the surface (Z) (its derivative) is zero.

Principal Axes. These are the axes on which the surface is said to rotate. Simply put: the lines of steepest and least steep ascend. The principal axes are the perpendicular lines that intersect at the stationary point and where at least one of the principal axes optimizes the amount of explained variance along its axis. The other principal axis subsequently, sometimes, minimizes the explained variance. If the surface is convex (peaks downward) the greatest ascend occurs along the first principal axis and the least occurs along the second principal axis. The reverse is true for concave surfaces (peaks upwards). In the case of a saddle point, both axes indicate paths of greatest ascend.

X = Y line. The points where two independent variables have equal value, i.e. perfect congruence. An important part of RSA is to examine the size of the slope on the surface along either the principal axes or the X = Y line and how close the principal axes and the X = Y line are to each other. For example, when perfect congruence between explicit and implicit self-esteem minimizes depression, and any incongruence adversely effects depression, the X = Y line and the principal axis would be identical. Finding the principal axes in a response surface can be difficult if one is unfamiliar with eigenbases. In the case of quadratic polynomial regression, which will be discussed in this paper, the easiest way to determine them is given in Edwards (1993).

RSA goes beyond linear regression. In linear regression, the mean of the dependent variable is modelled to be conditional on a linear set of independent variables. In its most basic form it constitutes an addition of main effects, which takes the form of:


5 \[
\widehat {Y} = {\beta _0} + {\beta _1}{X_1} + {\beta _2}{X_2} \cdots + {\beta _n}{X_n} + \epsilon
\]

Y being the value of the dependent variable, X representing the set of independent variables, Beta representing the regression weight with Beta-zero representing the intercept and epsilon to indicate normally distributed ~ *N*(0, σ^2^).

Sometimes linear regression contains a combination of main- and interaction effects. The addition of an interaction-term allows for the modelling of nonparallel lines when used with a categorical predictor, or for rotated flat surfaces when used on solely numerical predictors. Interaction implies that two variables have a multiplicative effect, which is why it is added to a regression model in the form of the multiplication between two variables, with a regression weight to determine the strength and direction of the multiplier. For example:


6 \[
\hat Y = {\beta _0} + {\beta _1}{X_1} + {\beta _2}{X_2} + {\beta _3}{X_1}{X_2} + \epsilon
\]

In polynomial regression, this regression model is expanded to allow for curved surfaces, which adds a new layer of complex ways in which variables are allowed to interact. The most basic polynomial regression is one in which the squared main effects are added to a model. It is possible to add higher-order polynomials to a model, and just like with standard linear regression the user is free to choose the form of the regression equation. In response surface methodology however the most parsimonious and widely used form is a variation of model (2):


7 \[
\hat Y = {\beta _0} + {\beta _1}{X_1} + {\beta _2}{X_2} + {\beta _3}X_1^2 + {\beta _4}{X_1}{X_2} + {\beta _5}X_2^2 + \epsilon
\]

Response surface analysis (RSA) was originally developed by Box and Wilson (1951) to determine optimal conditions for experimentation in a chemical investigation. Contrary to what social scientists usually call response, i.e. the score of a respondent on some variable, the *response* in response surface methodology refers to the set predicted scores on the criterion variable. The idea behind this was to optimize (to find the maximum or minimum) a set of responses, such as yield, purity or cost of the product, based on factors that influenced these responses, such as temperature and pressure. The advantage of modelling the interplay of the influential factors on the responses as a curved surface expressed by a simplified regression equation is that the region where changes in the factors would have the most pronounced effect can be efficiently determined.

**Figure 1:**
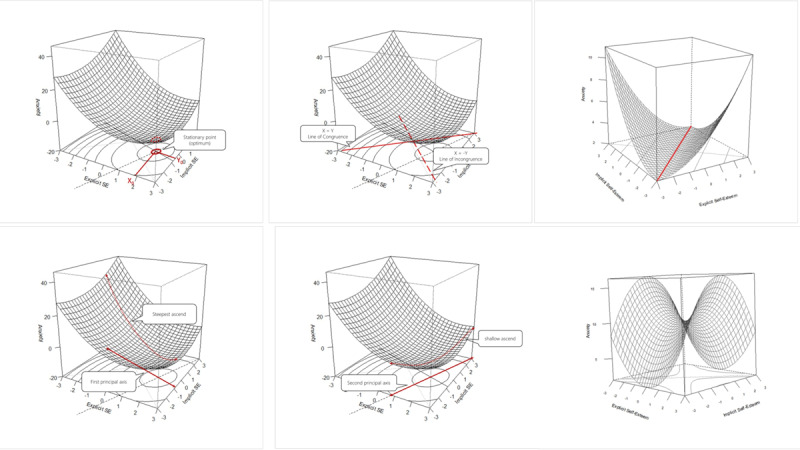
Six visualizations to explain RSA graphically.

In Figure 1, six visualizations are employed to elucidate key points of interest and patterns, arranged from left-to-right and top-to-bottom.

Figure a depicts a stationary point representing the optimum, where the values of implicit and explicit self-esteem converge to yield the lowest predicted anxiety.

Moving to Figure b, the congruence line (X = Y) and the incongruence line (X = –Y) are presented. In a hypothesis positing that congruency between predictors optimizes a given variable, one would anticipate the optimal anxiety value aligning with the X = Y line, with the least favorable values occurring along its inverse, the X = –Y line.

Figure c illustrates a hypothetical scenario of perfect congruence, where anxiety consistently is lowest when values of implicit and explicit self-esteem are congruent, accompanied by a rapid increase in anxiety when incongruence arises.

Figure d visualizes the relationship between the first principal axis and the steepest ascent, where movement along this axis at the base of the graph corresponds to a substantial change in anxiety. Figure e demonstrates how the second principal axis aligns with the second-largest change in anxiety, which is more gradual than the change along the first principal axis.

The final Figure f showcases a pattern known as a saddle point, challenging to interpret due to its stationary nature (it has a stationary point at the center of its ‘saddle’) without a clear optimum (lowest or highest value). Saddle points denote complex relationships; for instance, in Figure f, steepest increases in anxiety occur when implicit self-esteem is high and explicit self-esteem is low. Conversely, the direction of steepest descent points to low implicit self-esteem and high explicit self-esteem, suggesting that individuals with high implicit self-esteem and low explicit self-esteem are more prone to anxiety, while those with low implicit self-esteem and high explicit self-esteem are less likely to experience anxiety.

To demonstrate an RSA approach to difference score analysis, polynomial regression analysis was performed in R (version 3.6.1) with respectively depression, anxiety and aggression as dependent variables. Hierarchically explicit self-esteem, implicit self-esteem were added to the model. In step 1 the main effects were entered. In step 2 the quadratic terms were added, and the interaction between the main effects were added in step 3. Even though standardizing the predictors avoids multicollinearity in the interaction term, to avoid further multicollinearity between polynomials we calculated orthogonal polynomials with the poly() option in the lm() function in base-R (v4.2.2; R core team, 2022), which means that these parameters were scaled so that the second-order polynomial is orthogonal (i.e., uncorrelated) to the first-order polynomial. This has the advantage that it can be tested whether adding a second-order polynomial significantly improves the regression over the lower order.

## 4 Results

Prior to analysis, scores for depression, anxiety, aggression, explicit and implicit self-esteem were examined for accuracy of data entry, distributions, univariate and multivariate outliers. Two cases with extremely high z-scores (*z* > 3.29) on depression and one case with an extremely low z-score (*z* < –3.29) on explicit self-esteem were found to be univariate outliers. No cases were identified as multivariate outliers based on their Mahalanobis distance (*p* < .001). All three univariate outliers were winsorized, meaning that they were replaced with the first non-outlying value above or below it. Thirteen missing values in depression and anxiety, eleven in aggression, and nine in explicit self-esteem were found and imputed with predictive mean matching with the mice package (Van Buuren, 2011) (with ten mutations) based on the following variables: depression, anxiety, aggression, explicit self-esteem, and implicit self-esteem (D600 all, D600 practice, D600 trial).

The completed data from the research variables are summarized in Tables 1 and 2. Shapiro-Wilk’s tests for normality of distributions indicated that depression, anxiety, and aggression deviated from the normal distribution and were positively skewed (*p* < .001). Correlations between implicit self-esteem and other measures were close to zero and not significant (*p* > .05). Correlations between explicit self-esteem and other measures were negative and ranged from moderate (Depression *r* = –0.68, *p* < .01; Anxiety *r* = –0.60, *p* < 0.1) to weak (Aggression *r* = –0.27, *p* < 0.1).

**Table 1:** Means, standard deviations, and correlations with confidence intervals of all research variables after data imputation, *N* = 135.


VARIABLE	*M*	*SD*	1	2	3	4	5	6

1. Depression	4.22	3.77						

2. Anxiety	5.48	5.02	.78** [.71, .84]					

3. Aggression	2.79	22.42	.48** [.34, .60]	.57** [.45, .68]				

4. Explicit SE	21.20	5.06	–.61** [–.71, –.49]	–.57** [–.68, –.45]	–.24** [–.39, –.07]			

5. Implicit SE	0.28	00.37	.02 [–.15, .19]	.09 [–.08, .26]	–.05 [–.22, .12]	.01 [–.16, .18]		

6. contDif	–0.00	10.41	.45** [.30, .57]	.47** [.33, .59]	.13 [–.04, .29]	–.70** [–.78, –.61]	.70** [.61, .78]	

7. CreemersDif	1.11	00.87	.26** [.09, .41]	.29** [.13, .44]	.18* [.01, .34]	–.15 [–.31, .02]	–.07 [–.24, .10]	.05 [–.12, .22]


*Note*: *M* and *SD* are used to represent mean and standard deviation, respectively. Values in square brackets indicate the 95% confidence interval for each correlation. The confidence interval is a plausible range of population correlations that could have caused the sample correlation (Cumming, 2014). * Indicates *p* < .05. ** Indicates *p* < .01.

**Table 2:** Frequency counts for the two categorical difference score variables: categorical difference (congruent, damaged and fragile SE), and the dummy variable used in Creemer’s method (damaged and fragile). N = 135.


	CONGRUENT	DAMAGED	FRAGILE

Categorical	39	47	49

Creemer’s dummy	*NA*	67	68


## Analysis 1: Continuous Difference Scores

Three hierarchical multiple regressions were used to test the effect of the continuous difference score (implicit – explicit self-esteem) on standardized scores for depression, anxiety, and aggression. The results of the regression analyses are summarized in Table 3. The continuous discrepancy score between implicit and explicit self-esteem showed a positive and significant association with depression and anxiety. The difference score explained about 22.4% in depression, 22.6%, in anxiety, but only 2.6% in aggression. The discrepancy between implicit and explicit self-esteem, expressed as a continuous difference score indicates that depressive symptoms and anxiety increase change by circa one-third of a standard deviation when the discrepancy between implicit and explicit self-esteem increases by a whole point.

**Table 3:** The continuous difference score (z-implicit – z-explicit)and it quadratic term as orthogonal predictors of standardized depression, anxiety, and aggression. In block one the main effect is modelled, in the second block the quadratic term is added.


	DEPRESSION		ANXIETY		AGGRESSION	
		
	(1)	(2)	(3)	(4)	(5)	(6)

Difference (implicit – explicit SE)	0.339*** (0.055)	5.482*** (0.847)	0.340*** (0.055)	5.498*** (0.843)	0.115* (0.061)	1.851* (0.978)

Difference^2^		3.045*** (0.847)		3.146*** (0.843)		2.085** (0.978)

Intercept	0.000 (0.076)	–0.000 (0.073)	0.000 (0.076)	0.000 (0.073)	0.000 (0.085)	0.000 (0.084)

R^2^	.224	.293	.226	.299	.026	.058

Adjusted R^2^	.218	.283	.220	.289	.018	.044


*Note:* *p < 0.1; **p < 0.05; ***p < 0.01.

In a second step of the regression analysis, we added a quadratic term for the difference score. These quadratic terms were found to be statistically significant (*p* < 0.01), indicating curvilinear relationships. The models with quadratics terms showed markedly higher *R^2^*, namely 29.3% in depression, 29.9% in anxiety, and 5.8% in aggression.

For standardized depression, the positive coefficient of 3.045 suggests that as the difference score increases, reflecting a shift from lower explicit to higher implicit self-esteem, the rate of change in standardized depression initially rises until reaching a minimum point at –0.90. Subsequently, the rate of change decreases.

Similarly, in the case of standardized anxiety, the quadratic term yielded a positive coefficient of 3.146, signifying an analogous curvilinear relationship with a minimum point at –0.87. This suggests that the transition from lower to higher implicit self-esteem is associated with an initial increase in anxiety, peaking at –0.87 on the difference score.

Additionally, for standardized aggression, the positive coefficient of 2.085 indicates a curvilinear association with the difference score, peaking at –0.44. This implies that the shift from lower to higher implicit self-esteem is linked to an initial increase in aggression, reaching its minimum point at –0.44 on the difference score.

## Analysis 2: Categorical Difference Scores

Three hierarchical multiple regressions were used to test the effect of the categorical difference score (implicit – explicit self-esteem) on standardized scores for depression, anxiety, and aggression. The results of the regression analyses are summarized in Table 4. The three types of difference were split into two dummy variables. Kernis’ terms for these dummy variables (Kernis, 2003) were used for labelling the categories as damaged (I > E) and fragile (E > I) so that congruency was expressed as the intercept to test the two types of discrepancy against. Similar to continuous discrepancy, the categorical difference score shows a positive, albeit weaker relationship with, respectively, depression and anxiety. The categorical difference score explained about 16.6% in depression, 18.3% in anxiety, but only 1.9% in aggression.

**Table 4:** The categorical difference score (z-implicit – z-explicit) as predictor of standardized depression, anxiety, and aggression. Fragile (SD < –0.5) and damaged (SD > 0.5) are entered as dummy variables with congruent self-esteem (0.5 SD) as the intercept.


	DEPRESSION	ANXIETY	AGGRESSION

	(1)	(3)	(5)

Damaged (I > E)	0.804*** (0.198)	0.856*** (0.196)	0.320 (0.214)

Fragile (E > I)	–0.093 (0.196)	–0.075 (0.194)	0.063 (0.212)

Intercept	–0.241* (0.144)	–0.265* (0.142)	–0.131 (0.156)

R^2^	.166	.183	.019

Adjusted R^2^	.154	.170	.004


*Note:* *p < 0.1; **p < 0.05; ***p < 0.01.

The effect of the discrepancy between implicit and explicit self-esteem, expressed as three categories, i.e. damaged, congruent and fragile, differs from the effect of the continuous difference score. The overall pattern of a positive linear relationship is maintained, but indicates that the effect is mostly, if not only due to the positive effect of damaged self-esteem on depressive symptoms (*β* = 0.80; *p* < 0.001) and anxiety (*β* = 0.86; *p* < 0.001). Fragile self-esteem had no significant effect on the three outcome variables. Neither fragile nor damaged self-esteem had a significant effect on aggression. A damaged self-esteem (high implicit self-esteem combined with low explicit self-esteem) increased mean depression and anxiety by around two thirds of a standard deviation and fragile self-esteem (combination of low implicit self-esteem with high explicit self-esteem) decreased mean depression by almost a quarter of a standard deviation. The results suggest that a damaged self-esteem might be associated with a higher mean depression and anxiety, but that the mean is still below average, and that the effect of the discrepancy is largely linear, meaning that congruent self-esteem is associated with both higher and lower depression and anxiety than incongruent self-esteem, with fragile self-esteem (E > I) seeming most beneficial.

## Analysis 3: Mixed Categorical and Continuous Scores

The approach demonstrated by Creemers et al. (2012) was tested by using three hierarchical multiple regressions on standardized scores for depression, anxiety, and aggression. The absolute value of the difference between implicit and explicit self-esteem (I-E) and the direction of the difference (negative vs. positive) were added to the models. The results of the regression analyses are summarized in Table 5. Comparable to the categorical approach, this approach labels the continuous discrepancy as either damaged (difference is positive, i.e., I > E) or fragile (difference is negative, i.e., E > I), but instead of a congruent group it defines two incongruent groups who differ in strength of incongruency (with a strength of zero for a perfect congruency). So the definitions differ a little from the categorical and continuous approach, and tries to offer what Creemers (2012) considers the best of both models. Without an interaction between size and direction (models 1, 3, and 5), the direction of the difference was only significantly related to depression and anxiety. None of these effects remained significant after adding the interaction between size and discrepancy. The interaction between size and direction was significant for depression and anxiety, but not for aggression. The positive interaction term (Table 5, Model 2, 4, and 6) coupled with the negative main effect of the direction of the discrepancy indicates that the effect of the discrepancy on depressive symptoms and anxiety is stronger in the damaged group, than in the fragile group. The mixed difference score explained about 29,3% in depression, 30,3% in anxiety, and 5,8% in aggression.

**Table 5:** Creemers method for handling difference scores; The absolute value of the difference is entered as a continuous predictor, and the direction of the difference as a dummy variable (z-implicit – z-explicit) with fragile when <0, and damaged when >0. The two variables are predictors of standardized depression, anxiety, and aggression.


	DEPRESSION		ANXIETY		AGGRESSION	
		
	(1)	(2)	(3)	(4)	(5)	(6)

Size of SE Discrepancy	0.346*** (0.090)	0.629*** (0.089)	0.362*** (0.114)	0.619*** (0.843)	0.221** (0.099)	0.294** (0.133)

Direction SE Discrepancy 1 = Fragile (0 = Damaged)	–0.726*** (0.153)	–0.018 (0.240)	–0.766*** (0.151)	–0.124 (0.238)	–0.264 (0.169)	–0.081 (0.277)

Size * Direction		–0.644*** (0.173)		–0.584*** (0.172)		–0.166 (0.200)

Intercept	–0.020 (0.146)	–0.329** (0.162)	–0.017 (0.144)	–0.298* (0.161)	–0.112 (0.161)	–0.192 (0.187)

R^2^	.218	.293	.241	.303	.053	.058

Adjusted R^2^	.206	.277	.230	.287	.038	.036


*Note:* *p < 0.1; **p < 0.05; ***p < 0.01.

### Analysis 4: Polynomial Regression

**Figure 2:**
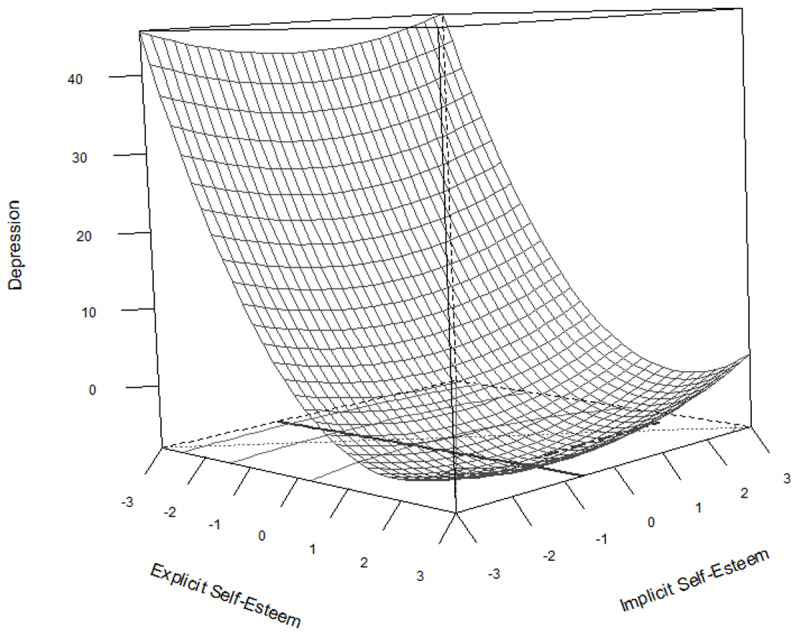
Response surface plot representing the regression equation for the relationship between explicit and implicit self-esteem on depression.

**Figure 3:**
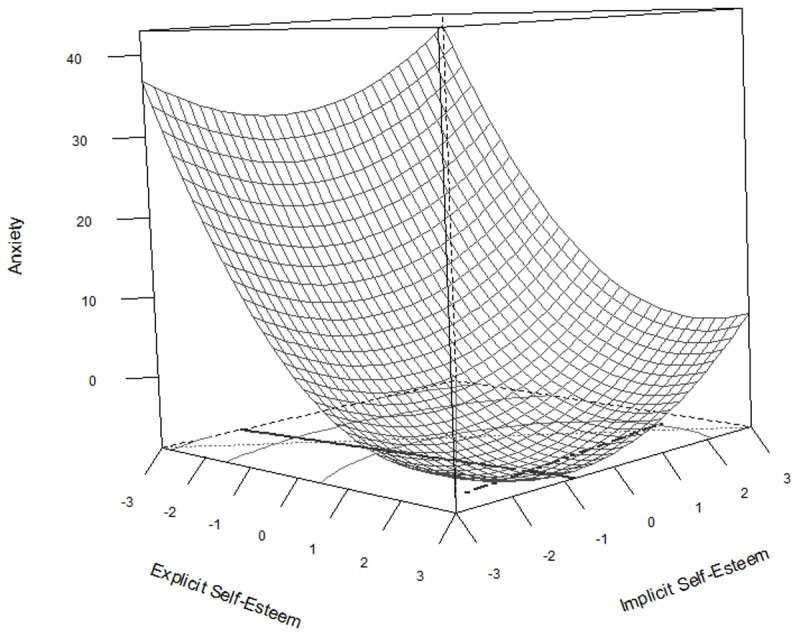
Response surface plot representing the regression equation for the relationship between explicit and implicit self-esteem on anxiety.

**Figure 4:**
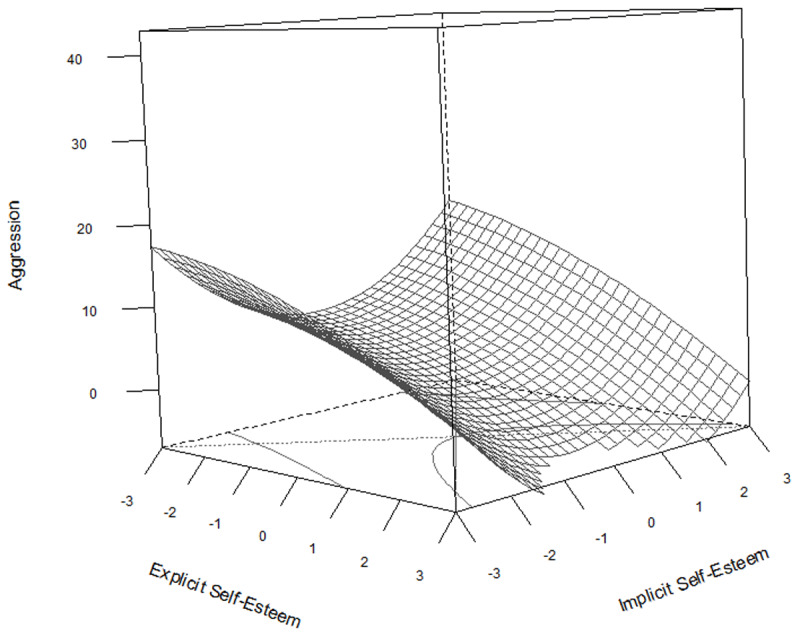
Response surface plot representing the regression equation for the relationship between explicit and implicit self-esteem on aggression.

The results of the regressions are summarized in Table 6 and visualized in Figures 2, 3, and 4.

**Table 6:** Polynomial regression of orthogonal factors based on the standardized predictors explicit and implicit self-esteem as predictors of standardized depression, anxiety and aggression. For each dependent variable three models are shown; the first contains only main effects, the second adds polynomial terms, and the final, third model adds an interaction term.


	DEPENDENT VARIABLE:								

	DEPRESSION			ANXIETY			AGGRESSION		
		
	(1)	(2)	(3)	(4)	(5)	(6)	(7)	(8)	(9)

Explicit self-esteem	–0.660*** (0.065)	–7.660*** (0.741)	–7.638*** (0.731)	–0.591*** (0.070)	–6.625*** (0.799)	–6.863*** (0.785)	–0.266** (0.083)	–3.169*** (0.974)	–3.149*** (0.969)

Implicit self-esteem	0.019 (0.065)	0.167 (0.739)	0.310 (0.733)	0.089 (0.070)	1.254 (0.801)	1.173 (0.787)	–0.037 (0.084)	–0.426 (0.972)	–0.291 (0.971)

Explicit self-esteem^2^		2.149*** (0.739)	2.232*** (0.731)		1.634 (0.799)	1.854** (0.785)		–0.009 (0.972)	0.069 (0.968)

Implicit self-esteem^2^		0.387	0.420		1.254	0.711		1.297	1.328

Explicit self-esteem * Implicit self-esteem			–0.135^**^ (0.064)			–0.170^**^ (0.069)			–0.127 (0.085)

Intercept	0.000 (0.065)	0.000 (0.064)	0.003 (0.063)	0.000 (0.070)	0.001 (0.069)	0.004 (0.067)	–0.000 (0.084)	–0.000 (0.084)	0.003 (0.083)

R^2^	0.435	0.471	0.488	0.355	0.380	0.410	0.073	0.085	0.101

Adjusted R^2^	0.426	0.454	0.468	0.345	0.366	0.387	0.059	0.057	0.066


*Note:* *p < 0.1; **p < 0.05; ***p < 0.01.

The relationship between explicit, and implicit self-esteem, and depression, respectively anxiety, and aggression is complex and non-linear. The numbers in Table 6 provide quantitative insights into this relationship, while Figure 2, respectively 3, and 4 visualizes it graphically. Figure 2 displays the regression equation for the relationship between explicit and implicit self-esteem on depression. The first principal axis can be understood as the path where the dependent variable changes the most, has its steepest ascend/descend. In Figure 2 the first principle axis is denoted by a thick black line at the floor of the graph. The shape of the surface is convex, with the steepest ascens running parallel to the x-axis, indicating that depression increases at an increasing rate when explicit self-esteem decreases, but is almost unaffected by changes in implicit self-esteem. The stationary point is located at X = 1.78 Y = –0.22, which indicates that depressive symptoms are lowest when explicit self-esteem is about one and a three quarters standard deviations above average, and increases slightly when explicit self-esteem increases beyond that point. Overall the surface indicates three effects. First, depressive symptoms decrease sharply when explicit self-esteem increases, but begins to increase again when explicit self-esteem exceeds about 1.75 SD. Second, depressive symptoms are relatively unaffected by implicit self-esteem, and are lowest when explicit self-esteem is high, and implicit self-esteem is around zero (the mean), and increase only lightly (and not-significantly) whenever implicit self-esteem is either high or low. Third, depressive symptoms are highest when explicit self-esteem is very low, and implicit self-esteem is very high (damaged), but close to the stationary point (lowest point) when explicit self-esteem is very high and implicit self-esteem is very low (fragile). Taken together with the regression coefficients in Table 6 the effects of the discrepancy between implicit and explicit self-esteem on depression seem almost solely driven by the significant main polynomial effect of explicit self-esteem, contrary to the conclusions of the earlier approaches.

Figure 2 visualizes the relationship between explicit, implicit self-esteem, and depression as response surfaces. The response surface for the model with only main effects is a plane, suggesting a linear relationship. As polynomial terms are introduced, the response surfaces become curved, indicating a non-linear relationship. The interaction term further enhances the curvature, representing the complexity of the relationship.

Figure 3 displays the regression equation for the relationship between explicit and implicit self-esteem and anxiety. The shape was convex with its stationary point located at X = 1.82 Y = –0.61, which indicates that anxiety was lowest when explicit self-esteem was just close to two standard deviations above average, and increases slightly when explicit self-esteem increases beyond that point. The slope of the first principal axis (steepest ascent) (*p*_11_ = –0.07) is close to zero, indicating that anxiety increases at an increasing rate when explicit self-esteem decreases, but is almost unaffected by changes in implicit self-esteem. The slope of the second principal axis was greater than 1 (*p*_21_ = 13.48) almost parallel to the Y-axis.

Overall the surface indicates three effects. First, anxiety decreases sharply when explicit self-esteem increases, but begins to increase again when explicit self-esteem exceeds about 1.8 SD. Second, anxiety is not-significantly affected by implicit self-esteem, and is lowest when explicit self-esteem is high, and implicit self-esteem is low, and increases only lightly (and not-significantly) whenever implicit self-esteem is either high or low. Third, anxiety is highest when explicit self-esteem is very low, and implicit self-esteem is very high (damaged), but close to the stationary point (lowest point) when explicit self-esteem is very high and implicit self-esteem is very low (fragile). Taken together with the regression coefficients in Table 6, the effects of the discrepancy between implicit and explicit self-esteem on anxiety seem primarily driven by the significant main polynomial effect of explicit self-esteem, which is contrary to the conclusions of the earlier approaches. Additionally, severely damaged self-esteem, i.e. a combination of extremely high implicit self-esteem and extremely low explicit self-esteem does seem to result in much higher anxiety than when there is a congruent extremely low explicit and explicit self-esteem, indicating that a discrepancy between explicit and implicit self-esteem has a stronger effect when explicit self-esteem is low, than when explicit self-esteem is high.

Figure 4 displays the regression equation for the relationship between explicit and implicit self-esteem on aggression. The only significant predictor of aggression in this model is explicit self-esteem. Overall the surface indicates two effects. First, aggression decreases very slightly and linearly when explicit self-esteem increases. Second, aggression is not significantly affected by implicit self-esteem, and is zero when explicit self-esteem is close to zero (average), and increases when it deviates negatively from zero. When considering the regression coefficients presented in Table 6 alongside the findings related to the incongruence between implicit and explicit self-esteem, notable parallels emerge with the results observed in method 1. In the latter, quadratic terms were introduced to the continuous difference scores, revealing significant relationships across all three dependent variables. However, the Response Surface Analysis introduces a nuanced perspective, suggesting that the continuous difference effect identified in method 1 is primarily explained by a significant main effect of explicit self-esteem, rather than implicit self-esteem or any interaction between the two.

## 5 Discussion

### 5.1 Discussion of results

The overall goal of this paper was to demonstrate the difference in results and associated conclusions between several frequently used approaches for testing theories of congruence between implicit and explicit measures. More specifically we contrasted three analyses based on discrepancy scores between implicit and explicit self-esteem (respectively a continuous discrepancy score, a categorical discrepancy score and a mixed categorical and continuous discrepancy score) with a polynomial regression approach (i.e., response surface analysis), and tested the association with respectively depressive symptoms, anxiety and aggression in a general population sample. Findings of the three analyses based on discrepancy scores resulted in conclusions that supported the theory that the incongruence between explicit and implicit self-esteem was associated with both depressive symptoms and anxiety. Furthermore, the analysis based on a categorical discrepancy score (analysis 2) and the analysis based on a mixed categorical and continuous discrepancy score (analysis 3) offered a more detailed conclusion, showing that damaged self-esteem (in which one experiences more implicit than explicit self-esteem) was associated with depressive symptoms and anxiety whereas fragile self-esteem (in which one experiences more explicit than implicit self-esteem) was not. As such, the implications of these results are that depressive symptoms and anxiety increase when the discrepancy between explicit and implicit self-esteem increases and in particular when explicit self-esteem is high and implicit self-esteem is low. Based on these findings one might conclude that measuring both explicit and implicit self-esteem in individuals seeking help for depressive symptoms and anxiety problems might be a good strategy to determine the specific focus of the psychological treatment in order to effectuate congruence between implicit and explicit self-esteem. However, the findings of the polynomial regression approach pointed toward a different conclusion and implication. In general, the polynomial regression showed that the relationship between depressive symptoms and anxiety was foremost driven by explicit self-esteem. Implicit self-esteem accounted for little of the variation in depressive symptoms and anxiety, and incongruence between explicit and implicit self-esteem seemed to become only relevant in extremes of explicit self-esteem. The latter conclusion though must be taken with caution as the number of individuals with extremes of explicit self-esteem was limited in this general population sample (see limitations). The overall conclusion of the polynomial regression approach that mainly explicit self-esteem and not implicit self-esteem is associated with depressive symptoms and anxiety suggests that measuring explicit self-esteem can offer relevant insights for the psychological treatment for individuals with depressive symptoms and anxiety problems. If explicit self-esteem is low, the main goal of the psychological treatment might be then to strengthen explicit self-esteem. All approaches were in agreement on the non-significance effect of congruency between implicit and explicit self-esteem on aggression. This might however leave one with the false impression that aggression was unaffected by either of the component elements of the difference score. Polynomial regression however showed a significant negative association between explicit self-esteem and aggression, but neither an association between implicit self-esteem and aggression, nor between the self-esteem discrepancy and aggression was observed. This suggests that explicit self-esteem rather than implicit self-esteem is important in explaining inter-individual variation in aggression. In sum, this study showed that conclusions regarding the role of implicit and explicit self-esteem and their discrepancy in outcomes such as psychopathological symptoms, can be misleading when congruency is modelled as a difference score between explicit and implicit measures. We would interpret the results as supporting the discrepancy hypothesis, whereas in fact there is only an effect of explicit self-esteem on depressive symptoms and anxiety. The results of the analyses based on the discrepancy scores basically reflect the main effect of explicit self-esteem. This was revealed by the results of the polynomial regression analyses. The combination of polynomial regression with response surface methods provided a more complete picture of the complex relationship between explicit and implicit self-esteem, leading to more precise and possibly more effective treatment suggestions. These findings are in concordance with previous work outlining the methodological issues associated with the use of discrepancy scores and proposing polynomial regression analysis and response surface methods as an alternative approach (Cafri et al., 2010; Humberg et al., 2018).

### 5.2 Limitations

Several limitations must be considered when interpreting the present results. Firstly, the study employed a cross-sectional design and therefore no conclusions can be drawn concerning the causal direction of the associations between the different forms of self-esteem and depressive symptoms, anxiety and aggression. Secondly, participants were recruited within the social networks of psychology students and, apart from variation in age, gender and education, most participants were in a job, study or retired, and involved in a relationship, limiting the generalizability of study findings to other groups, including individuals with disabilities, or patients diagnosed with a mental illness. Thirdly, implicit self-esteem was operationalized through the IAT using the D600 scoring algorithm recommended by Greenwald et al. (2003). However, Greenwald’s studies provide limited descriptions of the samples involved. It remains unclear how much the proposed thresholds by Greenwald et al. (2003) are valid in different populations, and it is likely that alternative thresholds should be explored for a new population. Since our study’s objective was to provide a practical example of employing polynomial analysis we have chosen to adhere to Greenwald et al. (2003) as closely as possible to provide a starting point in comparing methods. This decision means that the theoretical implications of the findings discussed here are contingent upon whether our sample was sufficiently similar to the one used by Greenwald et al. (2003) and what impact any differences might have on the results themselves.

Finally, this manuscript explores different ways to examine the effects of implicit and explicit self-esteem. Beyond just looking at the details, the differences among these approaches also affect how we think about and measure implicit and explicit self-esteem, which in turn influences how reliable and valid our analyses are. Implicit measures like the Implicit Association Test (IAT), dig into the spontaneous, context-dependent side of self-esteem by capturing automatic associations influenced by the immediate surroundings. Even though the IAT is less consistent over time compared to explicit measurements, it’s important to note that this doesn’t mean it’s not valid. The IAT is considered capable of genuinely capturing an aspect that is distinct from explicit measurement instruments, revealing parts of self-esteem that we might not consciously think about. In contrast, explicit measures, like the Rosenberg Self-Esteem Scale, involve a more intentional and reflective process similar to structured self-evaluation. The explicit approach relies on intentional, conscious introspection, which is quite different from the automatic, context-dependent nature of implicit measures. However, when we’re calculating difference scores we’re making assumptions about what self-esteem really means. The difference between the intentional, conscious nature of explicit measures and the automatic, context-dependent nature of implicit measures needs careful thought. This difference in assumptions highlights the importance of interpreting the results cautiously, especially when it comes to understanding how a gap between explicit and implicit self-esteem might affect things like depression, anxiety, or aggression. The main goal of the study is to show the effects of different analysis choices rather than providing a definite answer to these complex questions.

The data in this study reflect an important limitation regarding the interpretation of the polynomial regression plots: surface response models become poor descriptors of data when it is high on measurement error and when meaningful effects are only present in the tails of the data-distribution. All regression models become increasingly unstable nearer the tails of the distributions and polynomial regression is no exception. In our demonstration this limitation is exacerbated by the illusion of precision in the visualization of the 3d-surface. Even though explained variance was high, explained variance in the range of 20% still leaves the model with a lot of residual variance; error which increasingly expands closer to the extremes. The plots are visualized in such a way to optimise comparisons between plots. The scale of –3 < *z* < 3 is sufficient for implicit self-esteem (min *z* = –2.19; max *z* = 2.82), but leaves much to extrapolation for the high end of explicit self-esteem (min *z* = –2.59; max *z* = 1.71). This leaves the conclusions regarding effects on the extremes of explicit self-esteem (*z* > 2) as an extrapolated possibility.

But, this is part of the raison d’être of response surface modelling as advanced by Box and Wilson (1951). The strength of this approach lies in determining the optimal areas for further experimentation and was not intended as a way towards a theoretical model in and of itself. The implications for this study are that in order to test the theory of congruence, further research should aim to find a sample of respondents who are high to extremely high on explicit self-esteem. Our models suggest that if congruency effects are to be found, they are likely (statistically) to be found in a subset of the population extremely high on explicit self-esteem for depressive symptoms and anxiety, but probably not for aggression. Effects on depression and anxiety, for people with explicit self-esteem within two standard deviations of the mean, seem mostly driven by their explicit experience of self-esteem. One final remark can be made towards the use of the IAT as the operationalization of implicit self-esteem. Although the IAT is by far the most used method to capture implicit processes, there are some criticisms about the validity, the sensitivity to experimental manipulations and contextual factors and the susceptibility to faking (see e.g., Fiedler et al., 2006; Gawronski et al., 2007). The IAT has generally been found to be valid when the implicit construct being measured has been subject to experimental manipulation (Perugini et al., 2007). In this study the IAT was used to measure implicit self-esteem without any manipulation of it. Polynomial regression has been shown to be very effective to test the dual systems congruency hypothesis, and it would be worthwhile to test the congruency hypothesis with either (a) methods of measuring implicit self-esteem that are valid outside of experimental manipulation (e.g. Creemers et al., 2012), or (b) to replicate this study, but administer the IAT after manipulating implicit self-esteem. It is therefore recommended to replicate these findings as soon as better indirect measures of self-esteem are available (Buhrmester et al., 2011).

### 5.3 Conclusion

To conclude, this paper demonstrated the difference in results and conclusions between the use of discrepancy scores and polynomial regression in testing associations between explicit and implicit self-esteem and outcomes of psychopathology. The combination of polynomial regression with response surface methods has the potential to evaluate more complex and more detailed hypotheses which are simply not possible using statistical approaches based on discrepancy scores and is therefore recommended for future research disentangling the role of explicit and implicit self-esteem in psychological outcomes.
